# The rice production practices of high yield and high nitrogen use efficiency in Jiangsu, China

**DOI:** 10.1038/s41598-017-02338-3

**Published:** 2017-05-18

**Authors:** Jiuxin Guo, Xiangyu Hu, Limin Gao, Kailiu Xie, Ning Ling, Qirong Shen, Shuijin Hu, Shiwei Guo

**Affiliations:** 1 Jiangsu Provincial Key Lab for Organic Waste Utilization, National Engineering Research Center for Organic-based Fertilizers, Jiangsu Collaborative Innovation Center for Solid Organic Waste Resource Utilization, College of Resources and Environmental Sciences, Nanjing Agricultural University, Nanjing, 210095 China; 2 College of Resources and Environment, Fujian Agriculture and Forestry University, Fuzhou, 350002 China; 3 Department of Plant Pathology, North Carolina State University, Raleigh, NC 27695 USA

## Abstract

To face the great challenges of ensuring food security and environmental sustainability, agricultural production must be improved by high yield and high resource utilization efficiency (HYHE). We recently addressed this challenge and evaluated yield potential by surveying 735 farmers in 2008–2012 and then conducting 6 rice field experiments in 2008–2013 with large demonstration areas in 2010–2013 aimed to actualize the HYHE in Jiangsu Province, China. The survey result showed that the averaged N rate, grain yield and N partial factor productivity (PFP_N_) of the farmers were 336.7 kg ha^−1^, 8131.8 kg ha^−1^ and 24.2 kg kg^−1^, respectively. Through controlling total N rates and adjusting the application timing, the yield and the PFP_N_ of optimal N managements (OPT) were increased by 5.9% and 37.6% with 31.4% reduction in N supply amounts for 6 experimental sites, and the yield increased by 5.6% for large demonstration areas compared with farmers’ fertilizer practices (FFP), respectively. In conclusion, although the soil properties of the different regions varied, HYHE could be achieved by regulating the N management practices, thus contributing to higher rice production and lower environmental costs from intensive agriculture in Jiangsu, China.

## Introduction

Global crop yields have continuously increased to meet population growth, and in China, improving crop production to meet the food demand is critical because of the huge population^1, 2^. Cheng *et al*.^3^ reported that rice production in China would have to increase by 14% by 2030 to meet the requirements of the growing population, but to maximize grain yield, farmers often use more N fertilizer than the minimum required for maximum crop growth^4–6^. In the rice production system in China, the amount of N fertilizer used (209 kg ha^−1^) has been found to be 90% greater than the global average^7^; in particular, approximately 300–350 kg N ha^−1^ has been applied in Jiangsu Province^8^. However, China is currently the world’s largest consumer of N fertilizer, accounting for 30% of global N consumption, with a low N use efficiency (NUE) of approximately 30–35% for rice^9, 10^ and high environmental risk^11–13^.

To obtain high rice yield and NUE, several N management models have been studied in certain areas; for example, site-specific N management (SSNM) practices, such as real-time N management (RTNM) and fixed-time adjustable-dose N management (FTNM), were developed to increase rice yield and NUE^9, 14^. Field evidence has shown that proper nutrient management during rice production can improve crop yield while reducing the N application rate and decreasing the environmental costs through integrated soil-crop system management (ISSM)^4, 15^. Chen *et al*.^7^ found that FFP increased rice yield from 7.0 t ha^−1^ to 8.5 t ha^−1^ by ISSM practices while reducing the N rate of 209 kg ha^−1^ to 162 kg ha^−1^ in China. Cereal production security and the pressures of resource and environmental management simultaneously require HYHE, which has become the goal of modern agricultural development in China^7, 9^. Our previous study demonstrated that by regulating crop yield parameters (i.e., panicles, grains per panicle, and grain weight), optimal N management (OPT) strategies significantly increased rice yield and NUE compared to farmers’ fertilizer practices (FFP)^8^.

Jiangsu (116°18′–121°57′ E, 30°45′–35°20′ N), the most developed economic and agricultural province in China, is located along the low-middle reaches of the Yangtze River and the eastern part of the country (Extended Data Fig. 1). The province is divided into three regions, south, central and north, according to the ecological conditions, which are determined by the Yangtze River and the Huaihe River across the south and north, respectively, and the economic levels, which are richer in the south and poorer in the north. Jiangsu accounts for 7.5% of the rice cultivation area in China and contributes 8.4% of the rice production; the rice yield per area and amount of fertilizer applied are the highest in the country^8, 9^. It is therefore very important to increase NUE to achieve sustainable rice yield, so the fertilization practices of the HYHE agricultural model could be applied in China.

Much effort has been directed towards establishing the difference between OPT and FFP in terms of nutrient supply (N rate, time and ratio), planting density, the use of high-yielding varieties and other management practices (irrigation, weeding) through one or two field experiments^16–18^. Furthermore, it is necessary to determine the current fertilization practices and conduct multi-location field experiments with large areas of application in specific agricultural production regions to evaluate the effect of OPT.

In this study, we firstly investigated the agricultural practices of the rice production in Jiangsu through a survey of 735 farmers. Then, 6 field experiments were conducted at different locations, and large demonstration areas were established. The objectives of this study were to (i) evaluate the N fertilizer input and distribution in Jiangsu, (ii) determine the feasibility of reducing the N application rate in Jiangsu without significant negatively affecting rice yield using multi-location field experiments with large application areas, and (iii) establish the optimal N management strategies to guide farmers to achieve HYHE.

## Results

### Agricultural situation in Jiangsu

The mean monthly air temperature and precipitation data for annual agricultural production from 2001 to 2013 are shown in Extended Data Fig. 2. A distinct, increasing trend was observed in both temperature and precipitation from January to July, which was then followed by a gradually decreasing trend from July to December. From rice transplanting (June) to the tillering (July) stage, in particular, maximum temperature and precipitation were observed as 275.4 mm and 29.2 °C, respectively. The mean monthly precipitation ranged from 32.2–275.4 mm during the period of rice growth (from June to November), and the mean monthly temperature showed high variation and ranged from 10.1 to 29.2 °C. Additionally, the climate data also showed significant differences between south, central and north Jiangsu. The mean annual temperature and precipitation from October to June were south > central > north, but the precipitation from July to September was north > central > south.

The properties of the basal soil were obtained from the China Soil Testing and Formulated Fertilization database for the period from 2010–2012 (Table 1). The soil properties among the three regions exhibited significant differences; the lowest organic matter content was found in the north of Jiangsu. Conversely, the lowest Olsen-P and pH were found in the south. Simultaneously, the total N showed a decreasing trend from the south to the north of Jiangsu. The mean properties of the top layer soil (0–20 cm) in Jiangsu were as follows: organic matter 23.33 g kg^−1^, total N 1.37 g kg^−1^, Olsen-P 14.76 mg kg^−1^, NH_4_OA_C_-K 118.21 mg kg^−1^ and pH 7.17. These results also apply to the distribution of basal soil properties in south, central and north JS (Extended Data Fig. 3).

**Table 1:** Physical and chemical properties of the basal soil (0–20 cm, n = 1050–1350) in south, central and north Jiangsu.

Location	Organic matter (g kg^−1^)	Total N (g kg^−1^)	Olsen-P (mg kg^−1^)	NH_4_OA_C_-K (mg kg^−1^)	pH (H_2_O)
South	24.73 a	1.61 a	12.95 b	89.51 c	6.54 b
Central	24.66 a	1.52 b	15.74 a	109.15 b	7.46 a
North	21.65 b	1.15 c	15.18 a	141.11 a	7.39 a

### Grain yield and N management under FFP

The rice planting areas gradually increased from south to north in Jiangsu. The average N rates, grain yield and PFP_N_ of FFP treatment were 336.7 kg ha^−1^, 8131.8 kg ha^−1^, and 24.2 kg kg^−1^, respectively, according to the survey of farmers in 2008–2012 (Table 2). The N application rate in central Jiangsu was 14.0% and 2.0% higher than that in the south and north of Jiangsu, respectively, and the N rates in both north and central Jiangsu was higher than the average level in Jiangsu. The variations in grain yield and PFP_N_ gradually declined from south to north in Jiangsu (Table 2).

**Table 2:** N rate, grain yield and N partial factor productivity (PFP_N_) in famer’s field in Jiangsu by the farmer survey.

Location	N rate (kg ha^−1^)	Grain yield (kg kg^−1^)	PFP_N_ (kg kg^−1^)
South (n = 157)	310.1 b	8537.2 a	27.5 a
Central (n = 190)	353.5 a	8041.6 ab	22.7 b
North (n = 389)	346.5 a	7816.6 b	22.6 b

**Figure 1:**
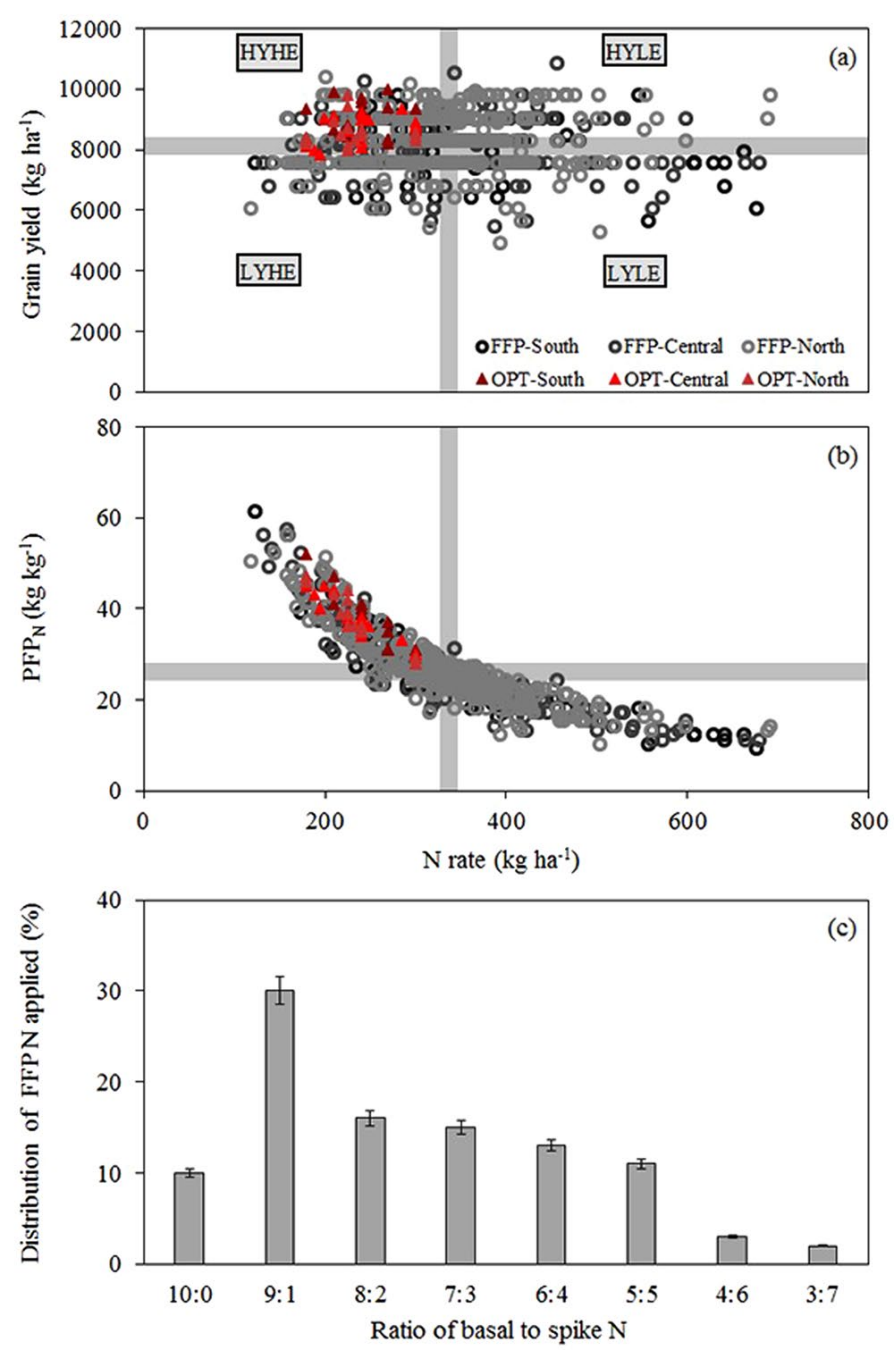
The distribution of yield (**a**) and N partial factor productivity (PFP_N_, **b**) on farmers’ fertilizer practices (FFP) and optimized N managements (OPT), and the distribution of FFP N applied on basal to spike N fertilizer application ratios (**c**) for rice production. The FFP values are from farmer surveys, and the OPT values are from the location experiment. The lengths of the bars (grey colour) are the average values plus 2% of the N rate, grain yield and PFP_N_. The short bars labelled HYHE, HYLE, LYHE and LYLE represent high yield and high efficiency, high yield and low efficiency, low yield and high efficiency, and low yield and low efficiency, respectively.

According to the classification of average yield and N rates, the average N rates, grain yield and PFP_N_ were 278.7 kg ha^−1^, 8900.6 kg ha^−1^ and 32.9 kg kg^−1^, respectively, but a frequency of only 27.2% was obtained under HYHE, indicating that a very small number of farmers could achieve HYHE (Extended Data Table 2, Fig. 1a). Approximately 23.1% farmers were classified as high yield and low efficiency (HYLE); they could achieve yields as high as 8966.1 kg ha^−1^ but consumed too much N at rates as high as 408.0 kg ha^−1^. The PFP_N_ decreased with increasing N rates (Fig. 1b), varying from the highest, 32.9 kg kg^−1^, in HYHE to the lowest, 17.9 kg kg^−1^, under low yield and low efficiency (LYLE) with an average of 24.2 kg kg^−1^ in Jiangsu. Moreover, as shown in our results, the basal to spike N fertilizer application ratio under FFP was imbalanced (Fig. 1c); 71% of the farmers adopted a basal to spike N fertilizer distribution ratios from 10:0 to 7:3, which were 2.5-fold higher than the distribution from 6:4 to 3:7 adopted by the rest.

### HYHE agriculture model (OPT) practices

As shown in Table 3, there was a significant positive effect of N application on grain yield and its components in rice, and the FFP and OPT yields were higher than the CK treatment. Compared with CK, N application could increase the average yield of 67.5%. Among the N management treatments, the maximum yield was 8.79 t ha^−1^ under the OPT treatment, and the yield was increased by 5.9%, which was greater than under the FFP treatment in the present study. The values of the yield components (panicles, grains per panicle, seed setting rate and 10^3^-grain weight) were also higher under the OPT treatment than FFP. However, the harvest index (0.51) of OPT increased slightly compared with FFP (0.49), and the smallest harvest index (0.46) was observed in CK. The above-ground biomass of FFP was higher than OPT at the tillering stage, but there was no significant differences between the flowering and harvest stages. These results were also vividly supported by the images collected during the location field experiment (Extended Data Fig. 4), and the yield and N rates of the OPT treatment could belong to the HYHE quadrant according to the results of the farmer survey (Fig. 1). Moreover, the NUE of TU_N_, PFP_N_, AE_N_, and RE_N_ exhibited significant differences under different N management models (Table 4); the highest NUE was always in the OPT treatment. Compared with FFP, the yield and NUE increased substantially although the N rates in OPT were decreased by 31.4%. Furthermore, the PFP_N_ of the OPT treatment in the location field experiment was always higher than the average PFP_N_ of the FFP treatment from the farmer survey (Fig. 1).

**Table 3:** Average biomass, yield components and harvest index of N managements across six sites in Jiangsu in 2008–2013.

Treatment	Above-ground biomass (t ha^−1^)			Panicles (×10^4^ ha^−1^)	Grains per panicle	Filled grain rate (%)	10^3^-grain weight (g)	Grain yield (t ha^−1^)	Harvest index
	Tillering	Flowering	Harvest						
CK	2.7 c	8.5 b	11.0 b	194.1 b	113.3 c	97.8 a	27.1 a	5.10 c	0.46 b
FFP	4.6 a	11.8 a	17.0 a	303.1 a	138.5 b	96.3 b	25.7 b	8.30 b	0.49 ab
OPT	3.6 b	12.2 a	17.3 a	290.7 a	151.8 a	97.1 a	26.5 a	8.79 a	0.51 a

**Table 4:** Average N rate, total N uptake (TU_N_), N partial factor productivity (PFP_N_), N agronomic efficiency (AE_N_) and N recovery efficiency (RE_N_) of N managements across six sites in Jiangsu in 2008–2013.

Treatment	N rate (kg ha^−1^)	TU_N_ (kg ha^−1^)	PFP_N_ (kg kg^−1^)	AE_N_ (kg kg^−1^)	RE_N_ (%)
CK	0	98.2 b	—	—	—
FFP	350	224.3 a	23.7 b	9.1 b	36.0 b
OPT	240	232.9 a	36.6 a	13.7 a	56.1 a

**Figure 2:**
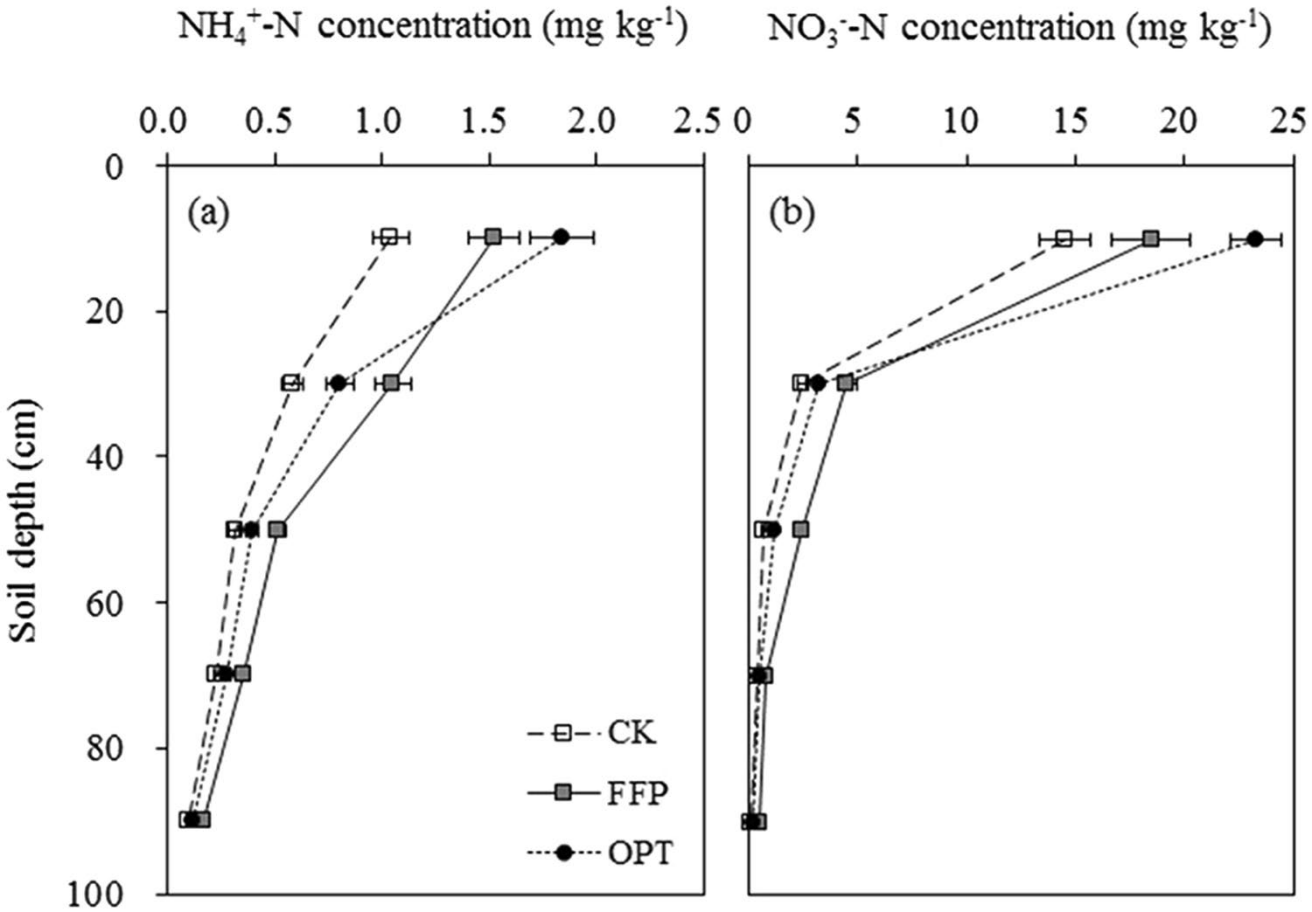
Distribution of NH_4_^+^-N (**a**) and NO_3_^−^-N (**b**) concentrations in the profiles of 0–100 cm soil depths at rice harvest in the Rugao site in 2013.

Our results also showed that the root morphology parameters significantly varied among the different N management practices. The parameters of root length, surface area, volume and number of root tips followed the general trend of CK > OPT > FFP, but the average diameter was OPT > FFP > CK at the seedling stage 10 days after transplanting (Table 5, Extended Data Fig. 5). Besides, the distributions of NH_4_^+^-N and NO_3_^−^-N concentrations in the profiles of different soil depths were significantly different between the types of N management at rice harvest in Rugao in 2013 (Fig. 2), the NH_4_^+^-N and NO_3_^−^-N concentrations were reduced with increasing soil depth. And the NH_4_^+^-N and NO_3_^−^-N concentrations under the OPT treatment were higher than under the FFP treatment in the top 20 cm of the soil layer, indicating that soil NH_4_^+^-N and NO_3_^−^-N were gradually leached down into deeper soil layers due to precipitation and irrigation.

**Table 5:** Root length, superficial area, average diameter, root volume and number of root tip of N managements of rice roots at the seedling stage 10 days after transplanting in Rugao sites in Jiangsu.

Treatment	Root length (cm plant^−1^)	Surface area (cm^2^ plant^−1^)	Average diameter (mm)	Root volume (cm^3^ plant^−1^)	Number of root tip (plant^−1^)
CK	481.7 a	50.6 a	0.34 b	0.43 a	4459.7 a
FFP	274.4 c	32.5 c	0.38 ab	0.31 b	1874.6 c
OPT	296.1 b	39.0 b	0.42 a	0.41 a	3047.0 b

**Figure 3:**
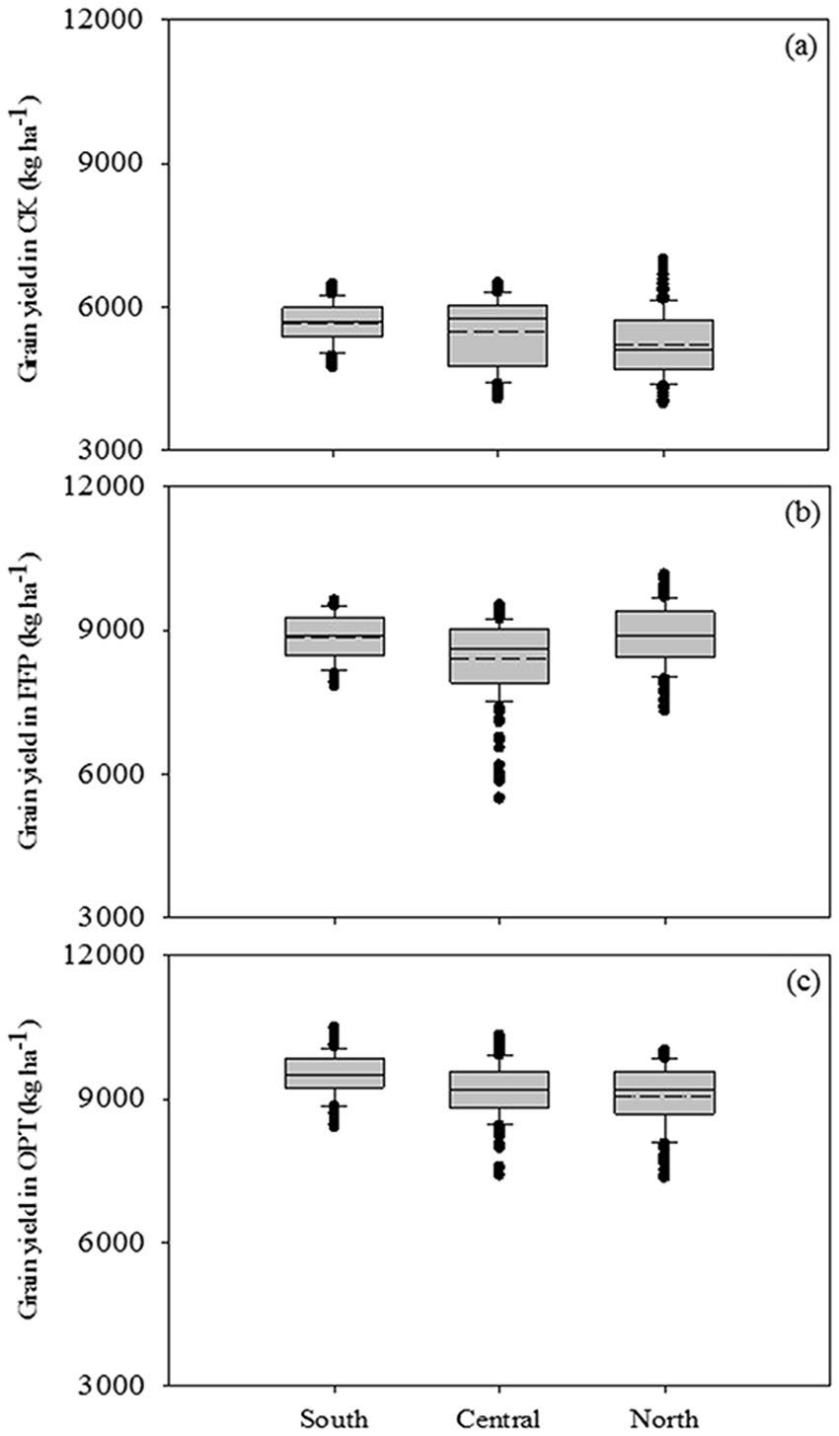
Rice grain yield of large demonstration areas on free-N control (CK, **a**), farmers’ fertilizer practices (FFP, **b**) and optimized N managements (OPT, **c**) in south, central and north Jiangsu in 2010–2013.

We also simultaneously analyzed the results from the large demonstration area under field conditions from the Soil Testing and Formulated Fertilization database for Jiangsu, and the results were consistent with the location field experiment, which showed a trend in rice yield of OPT > FFP > CK. The average OPT yield was increased by 5.6% compared with FFP (Fig. 3).

**Figure 4:**
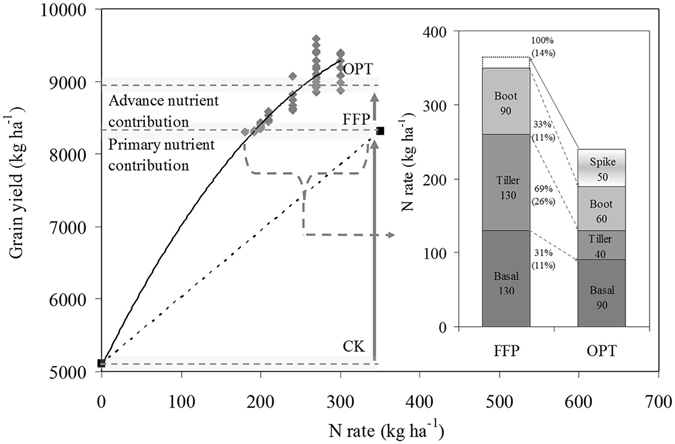
Response curves of rice grain yield to N rate and the nutrient contribution stage; further details are in Sui *et al*.^8^. The result of improving rice grain yield and nitrogen use efficiency depended on the optimized N managements (OPT, insert figure) at different rice growth stages. The insert figure shows rice N management strategies between OPT and farmers’ fertilizer practices (FFP). The percentage and the dotted and solid arrows represent the N rate ratios of reduced or increased between OPT and FFP for the different and total growth stages in and out of parentheses, respectively.

Based on the results of the location experiment and the large demonstration area experiment, the response curve of rice yield to N rates was generated, which revealed the existence of a nutrient contribution stage with different contributions of the N application rate to rice grain yield (Fig. 4). Compared with FFP in present the study, the OPT could achieve an advanced nutrient contribution stage by regulating the N rate, time and ratio.

## Discussion

As the most important cereal production system, intensive rice cultivation is prevalent in the Yangtze River Basin of China. Farmers typically apply excess N fertilizer in Jiangsu^4^, but some results have shown that rice yield under FFP was not always improved or even decreased under high N input^19^. Previous studies demonstrated that some OPT models improved rice yield by regulating the N fertilizer application rate and time in experts test^4, 8^. Therefore, to determine the best strategies to increase and maintain rice productivity in Jiangsu, it remains necessary to investigate the yield gap between OPT and FFP based on the farmers level.

In this study, the average fertilizer N rate of farmers’ reached 336.7 kg ha^−1^ from 2008–2012 in Jiangsu (Table 2). The N rates of FFP in south (310.1 kg ha^−1^), central (353.5 kg ha^−1^) and north (346.5 kg ha^−1^) Jiangsu were, respectively, 41.7%, 47.3% and 44.4% higher than the average N rate (240 kg ha^−1^) of our 6 field experiments, in which the N rates ranged from 180 to 300 kg ha^−1^ (Tables 2 and 4). Compared with FFP, the average N rates of OPT decreased by 31.4%, but rice yield was not reduced or even increased by 5.9% in the 6 field experiments and by 5.6% in the large demonstration area experiments (Table 4; Fig. 3), indicating excessive and unreasonable N application did not improve the yield. Since that all of the data of rice yield were obtained at maturity stage in October, and N use amount represented the total amounts during the whole growth stage, which was from June to October in Jiangsu province in China. Thus, the variability among the farmers’ fields were not likely resulted from the cultivation seasons. A similar result was obtained in a previous study^8^, and when the yield plateaued, increasing the nutrient level did not result in yield improvement because nutrients are no longer one of the main limiting factors^14^. The rice yield and TU_N_ of CK were also very high in our study (Table 3; Fig. 3), which suggested a higher soil N capacity at Jiangsu due to the higher nutrient accumulation in the soil from long-term excessive N fertilizer application. The grain yield gradually declined from south to north as revealed by both the results of the farmer survey and large demonstration area experiments (Table 2; Fig. 3), these results could be related to the soil properties of the different Jiangsu regions in that organic matter and total N content were also gradually reduced (Table 1; Extended Data Fig. 3). Therefore, N will be raised by an appropriate amount according to the status of the soil properties under OPT rice production. It is well established that the optimum N application rate for rice should depend on the soil fertility level^20–22^, and our results were basically similar to previous results concerning target yield and the recommended N application rate in an unfertilized, barren area. Some related reports recommended the appropriate amount of fertilizer for the different regions of Jiangsu, Deng *et al*.^23^ suggested that an N application rate of 150–200 kg ha^−1^ was the best for achieving a rice grain yield of 7600–8300 kg ha^−1^ and a decrease in the N loss of 110 kg ha^−1^ into Taihu Lake in the south of Jiangsu; Sui *et al*.^8^ found that a high rice yield (ranging from 9000 to 9400 kg ha^−1^) can be achieved with 180–300 kg N ha^−1^, and significantly improved NUE compared to the FFP with 255–350 kg N ha^−1^ in Jiangsu. The mineral N content in 0–90 cm soil profiles were frequently found to be between 50–100 kg N ha^−1^ after the winter wheat harvest in the Taihu Lake area^24^, indicating that a high level of residual mineral N existed in the plow layer and the potential that reduced N fertilizer could maintain high crop production.

As one of the most important indices for cereal crops, PFP_N_ directly reflects N input and economic income. Combined data from different areas of China showed that the average PFP_N_ of FFP was 41 kg kg^−1^ with 209 kg N ha^−1^ in rice fields^7^, and the average PFP_N_ of FFP from 26 rice field experiments in the Taihu Lake area was 26.7 kg kg^−1^ with 300 kg N ha^−1^ in Jiangsu^4^. In the current study, the average PFP_N_ of FFP was 24.2 kg kg^−1^ according to the farmer survey or 23.7 kg kg^−1^ from the location field experiment, which were both significantly lower than the OPT value (36.6 kg kg^−1^) in Jiangsu. The lower PFP_N_ of FFP generally occurred due to the supply model used by the farmers, that most of the N is applied to rice at the early vegetative stage. The basal to spike N fertilizer distribution ratio was from 10:0 to 7:3 for 71% of the farmers, while 29% employed a distribution ratio from 6:4 to 3:7 according to the farmer survey (Fig. 1c). These results agreed with the studies by Sui *et al*.^8^ and Peng *et al*.^9^, who found that approximately 60–70% of the total N was applied in the first 10 days after transplanting in the FFP of Jiangsu. The high fertilizer N input and the improper N application timing were important factors resulting in low AE_N_. Peng *et al*.^9^ reported that the AE_N_ of FFP was 3.6 kg kg^−1^, whereas the AE_N_ of the modified FFP, a 30% reduction in the total N application rate during the rice vegetative stage, was 7.7 kg kg^−1^ at sites in China. In the present study, the average AE_N_ of the FFP was 9.1 kg kg^−1^ in the 6 location experiments during 2008–2013 (Table 4), which was consistent with Sui *et al*.^8^ and Peng *et al*.^9^, and the NUE (TU_N_, PFP_N_, AE_N_, RE_N_) was significantly different between N management models with the OPT treatment values always higher than the FFP values (Table 4). Additionally, the FFP treatment resulted in a weaker and smaller root system compared with OPT (Table 5; Extended Data Fig. 5), indicating that the nutrient uptake capacity was reduced and the lodging risk was increased, while a stronger and larger root system in OPT could be beneficial for reducing N losses at the early vegetative growth stage of rice due to the massive amount of N being applied^25, 26^. These results suggested that a reduction in the proportion of N applied at the early vegetative stage and an increase in the proportion applied at the later reproductive growth stages benefited grain yield and NUE. Therefore, a valuable N management strategy was established by controlling total N rates and adjusting the application timing to improve crop production, and this made it possible to close the yield potential and gaps between OPT and FFP for the HYHE model in our experiments.

There are three processes that occur when N fertilizer is applied to the soil. Firstly, the fertilizer is absorbed and used by the crop plants; secondly, it is fixed by the soil; and thirdly, the fertilizer residues in the soil are lost from the paddy field into the environment. Ammonia volatilization, denitrification, and nitrate leaching are the main pathways of nitrogen loss^27^, and in Jiangsu, farmers often applied a higher amount of N fertilizer to maximize the grain yield in rice-based cropping system, which resulted in a lower NUE because of rapid N losses to water (surface and ground water) or the atmosphere^28, 29^. Ammonia volatilization and N_2_O emission constituted a very large proportion of the N lost from the paddy soil system, accounting for 10%–60% of the total N applied^7, 13, 30^.

The Mei-yu season always occurs in June and July, and it generally begins in mid-June in the Yangtze River Basin, bringing high precipitation, the amount of which determines the drought or flood conditions in the Yangtze River Basin^31^. Nitrate is one of the most water-soluble anions, and it can be easily leached into the groundwater system^16^. Excessive N fertilizer application to the soil has been identified as a major source of groundwater nitrate contamination^32, 33^, and our results showed that large amounts of N were applied as basal and tillering fertilizers for FFP rice production around the Mei-yu season when precipitation and temperature reach peak yearly values in Jiangsu (Extended Data Fig. 2), which is the main reason for N loss. In the past two decades, nitrate concentrations in the rivers and lakes of the Taihu watershed of Jiangsu have increased fivefold^34, 35^, and the nitrate content of well water in this region has ranged from 0.1 to 23 mg N L^−1^ with 41% of the drinking water exceeding the safety criterion of 10 mg N L^−1^. Qiao *et al*.^19^ and Liang *et al*.^36^ showed that (i) the nitrate concentration in leachate increased with the increase in N application rates; (ii) higher potential amounts of leachate would be engendered with a precipitation intensity of more than 5.9 mm d^−1^; (iii) the nitrate concentrations in leachate could increase with the increase of air temperature. These reasons could also contribute to an increase in ammonia volatilization and N_2_O emission in the uplands and lowlands^28, 37, 38^. Our results indicate that the NUE of rice plants can be easily increased by OPT by applying nearly 31% less mineral N fertilizer compared to the FFP, resulting in a significant increase in grain yield (Tables 3 and 4). Furthermore, the soil NH_4_^+^-N and NO_3_^−^-N were gradually leached into the deeper soil layers, and the leaching potential of the FFP treatment was greater than that of the OPT treatment in the 20–100 cm soil layers (Fig. 2). Therefore, we can estimate that the N losses to the environment can be efficiently decreased, while rice production can be maintained at a high level by reducing the overall N application rate with optimal N management^6, 7, 12, 39^.

To determine the optimal N application rates for rice production, it was necessary to consider the production, economic and environmental benefits^7, 40^. Our results showed that OPT significantly improved grain yield and NUE in all of the 6 location field experiments and the large demonstration area experiments (Tables 3 and 4; Fig. 3), which can be attributed to the integration of the following technologies: (i) the determination of SSNM to control the N application rate according to the China Soil Testing and Formulated Fertilization database for nutrient imbalances^14, 41, 42^; (ii) the RTNM technology to apply less basal N fertilizer and more panicle fertilizer^9, 43^; (iii) the ISSM strategy to achieve the best fertilizer management practices including fertilizer types, deep application, crop straw return and varieties with high nutrient efficiency^7, 15, 44^; (iv) other management practices including planting density, irrigation measures, integrated pest management, etc.^17, 45, 46^. In addition, the awareness of how to persistently improve soil fertility and productivity must always be incorporated into agricultural practices^2, 47^. These technologies are currently recommended in China due to their positive effect on increasing grain yield, reducing N loss and improving NUE, while simultaneously achieving improved nutrient contributions under OPT compared to FFP^7, 41, 48, 49^.

In conclusions, our results showed that higher fertilizer N input rate and improper timing practiced by farmers are becoming widespread, result in lower yield and NUE in rice production in Jiangsu, China. The HYHE agriculture model based on OPT strategies were conducted with a 31% reduction in the N application rate, achieving a 6% higher rice yield and 38% higher PFP_N_ even when the soil properties in the different regions of Jiangsu were various. Our research revealed that the OPT could achieve an advanced nutrient contribution stage based on FFP by regulating the N supply amount and time. The integration of appropriate nutrient management strategies with related agronomic practices are the keys to obtaining more productive, profitable and environmentally sustainable crop management practices.

## Methods

### Climate data collection

Data on the mean monthly air temperature and precipitation required for annual agricultural production in Jiangsu for the period 2001–2013 were obtained from the China Statistical Yearbook, 2002 to 2014, online database (www.stats.gov.cn) and are presented in Extended Data Fig. 2.

### Farmer survey

Surveys of FFP were carried out from 2008–2012, according to a random sampling method. Based on the differences in geographic location, climatic conditions and the level of regional economic development level, 30 counties were selected. Within each county 2 to 3 towns were randomly selected; 2 to 3 villages were selected in each town, and 8 to 10 farmer households were selected in each village. In total, 735 valid questionnaires about rice planting time, fertilizer utilization amount (including N, P, K fertilizer and also microelement fertilizer), tillage method, irrigation amount and time, the utilization of pesticide each year were obtained, and the numbers from the south, central and north Jiangsu were 157, 190 and 389, respectively. The respondents were visited and questioned by researchers to collect information about the use of chemical fertilizers in each household^7^.

The physical and chemical properties of the 0–20 cm soil layer from south, central and north Jiangsu for the period of 2010–2012 were obtained from the Soil Testing and Formulated Fertilization database (so called “3414 Program”, n = 1050–1350) of the Ministry of Agriculture, China.

### Description of the N management model experiments

Six different field experiments were conducted to test the N management models in the three regions of Jiangsu from 2008 to 2013. The counties of Changshu and Liyang, Rugao and Xinghua, and Donghai and Xinyi were selected to represent the south, central and north regions of Jiangsu, respectively (Extended Data Fig. 1). The experimental design followed the “3 + X” approach with three different N management models (Extended Data Table 1): (1) CK, free-N control; (2) FFP, in which a total amount of 350 kg ha^−1^ N was divided into 130, 130, and 90 kg ha^−1^ N and applied at the basal, tillering, and panicle initiation stages, respectively; (3) OPT, in which an N application rate from 180–300 kg ha^−1^, with an average of 240, was applied at the ratio of 38: 17: 25: 20 at the basal, tillering, panicle initiation, and spikelet differentiation stages, respectively, and this application method was adopted based on the results of published articles by Sui *et al*.^8^ and Peng *et al*.^9^. “X” represented the improved OPT based on the regulation of N application rate, time and ratio compared with FFP. Phosphorus (75 kg P_2_O_5_ ha^−1^) was applied as a base fertilizer, and potassium (90 kg K_2_O ha^−1^) was equally divided between the basal and panicle initiation stages. The OPT treatment was also defined as the HYHE agricultural model. All treatments were arranged in a randomized block design with four replications in fixed plots from 66.7 m^2^ to 90 m^2^ in size. The rice transplanting intensity was 30.77 × 10^4^ hills ha^−1^, and the hill spacing was 25 cm by 13 cm. Thirty-day-old seedlings were transplanted in from June 20–30 of each year, and field management and irrigation followed local high-yielding practices. Insects were intensively controlled using chemicals to avoid biomass and yield losses.

The rice grain yield data from large demonstration areas with different N management models (CK, FFP, and OPT) in Jiangsu for the period 2010–2013 were obtained from the Soil Testing and Formulated Fertilization database (3414 Program, n = 230–300) of the Ministry of Agriculture, China.

### Image collection

All digital images of rice plants were obtained in 2013 using a digital camera (EOS Kiss X50, Canon, Ota, Tokyo, Japan) at three different growth stages: tillering, flowering and harvesting.

### Sampling and laboratory procedures

At different growth stages (tillering, flowering and harvest), three rice plant hills of about 0.1 m^2^ with 20–30 plants each were sampled for biomass and mineral elements analysis. All samples were washed briefly with both tap and distilled water. The dry weights of all rice plant samples, including the different organs (leaves, stems, sheaths and spikes), were determined after drying in an oven at 105 °C for 30 min and then at 70 °C to a constant weight. At harvest, a 5 m^2^ (5.0 × 1.0 m) micro-plot in the middle of each plot were harvested manually for avoiding edge effects to determine grain yield and yield components. Moreover, the grain yield was expressed on a wet mass basis with a moisture level of approximately 15%.

To determine plant N content, dried and ground samples were digested with H_2_SO_4_-H_2_O_2_ at 260–270 °C and then analyzed with an Auto Analyzer 3 digital colorimeter (AA3, Bran + Luebbe, Norderstedt, Hamburg, Germany). Measurements were checked using certified standard reference materials obtained from the Institute for Environmental Reference Materials of the Ministry of Environmental Protection (Beijing, China)^50^. The data parameters were calculated by the following equations: harvest index (HI) = grain yield/above-ground biomass, total N uptake (TU_N_, kg N ha^−1^) = above-ground biomass × plant N content, N partial factor productivity (PFP_N_, kg grain kg N applied^−1^) = grain yield with N application/applied N rate, N agronomic efficiency (AE_N_, kg grain kg N applied^−1^) = (grain yield with N application - grain yield without N application)/applied N rate, N recovery efficiency (RE_N_, kg N kg N applied^−1^) = (total N uptake with N application - total N uptake without N application)/applied N rate.

In 2013, five soil cores containing the 0–100 cm soil layers were collected from each plot using an auger (inner diameter of 3.0 cm) at rice harvest in the Rugao experimental site in central Jiangsu. The soils from each 20 cm interval throughout the 0–100 cm layers were collected separately, and the soil from the same layer and plot was mixed. The fresh soil samples were thoroughly composited, and 12 g sub-samples were extracted using 100 ml of 0.01 mol L^−1^ CaCl_2_ and shaken for 1 h^51^. After filtration, the extracts were immediately measured for NH_4_^+^-N and NO_3_^−^-N concentrations with an Auto Analyzer 3 digital colorimeter (AA3, Bran + Luebbe, Norderstedt, Hamburg, Germany). The soil NH_4_^+^-N and NO_3_^−^-N concentrations were expressed on the basis of the oven-dried soil.

### Determination of root morphology parameters

Ten days after transplanting, the rice seedlings were collected from the N fertilizer management model experiment in Rugao site in 2013, and the samples were carefully cleaned on a 0.5 mm mesh screen. After removing the debris, an 8-bit grey scale image was acquired by digital scanning at a resolution of 600-dpi using an image scanner and a positive film transparency unit (Epson Expression 10000XL, Epson America, San Jose, CA, USA). All of the images of rice roots were analyzed to assess the morphology parameters using WinRHIZO (Version 2009b, Regent Instruments, Montreal, QC, Canada) in Lagarde’s mode^52^.

### Data analysis

All data were analyzed by ANOVA with the SAS 9.0 statistical software package (SAS Institute, Cary, NC, USA). The means of the treatments were tested using the least significant difference (LSD) test at the 0.05 significance level.

## Electronic supplementary material

Supplementary information
