# Supplementary material for: The rice production practices of high yield and high nitrogen use efficiency in Jiangsu, China
Source: Sci Rep. 2017 May 18;7:2101. doi: 10.1038/s41598-017-02338-3 (PMC5437039; doi:10.1038/s41598-017-02338-3)
Supplement: Supplementary file 1 — Supplementary information [file 41598_2017_2338_MOESM1_ESM.pdf]

The rice production practices of high yield and high nitrogen use efficiency in Jiangsu, China

Jiuxin Guo[1,2,*], Xiangyu Hu[1,*], Limin Gao[1], Kailiu Xie[1], Ning Ling[1], Qirong Shen[1], Shuijin Hu[1,3] & Shiwei Guo[1]

[1] Jiangsu Provincial Key Lab for Organic Waste Utilization, National Engineering Research Center for Organic-based Fertilizers, Jiangsu Collaborative Innovation Center for Solid Organic Waste Resource Utilization, College of Resources and Environmental Sciences, Nanjing Agricultural University, Nanjing 210095, China

[2] College of Resources and Environment, Fujian Agriculture and Forestry University, Fuzhou 350002, China

[3] Department of Plant Pathology, North Carolina State University, Raleigh, NC 27695, USA

[*]These authors contributed equally to this work.

Correspondence and requests for materials should be addressed to S.G. (email: sguo@njau.edu.cn)

Supporting Information

Extended Data Table 1 | The amount of N fertilizer applied (kg ha^{-1}) to N managements at different growth stages in the experiment conducted at Jiangsu in 2008-2013.

Treatment	CK	FFP	OPT		
			OPT1	OPT2	OPT3
Total N	0	350	180 - 240	270	300
Basel fertilizer	0	130	68-91	102	114
Tillering fertilizer	0	130	31 - 41	46	51
Panicle initiation	0	90	45 - 60	68	75
Spikelet differentiation	0	0	36 - 48	54	60
Total P_2O_5	75	75	75	75	75
Basal fertilizer	75	75	75	75	75
Total K_2O	90	90	90	90	90
Basal fertilizer	45	45	45	45	45
Panicle initiation	45	45	45	45	45

CK: free-N control; FFP: farmers' fertilizer practices; OPT: optimal N managements.

Extended Data Table 2 | The grain yield and PFP_N under high yield and high efficiency (HYHE), high yield and low efficiency (HYLE), low yield and high efficiency (LYHE) and low yield and low efficiency (LYLE), according to the average FFP yield and N rate in Jiangsu as defined by the farmer survey.

Classification	Average N rate (kg ha^{-1})	Average yield (kg ha^{-1})	N rate (kg ha^{-1})	Grain yield (kg ha^{-1})	PFP_N (kg kg^{-1})	Frequency (%)
HYHE	<336.7	>8131.8	278.7 b	8900.6 a	32.9 a	27.2
HYLE	>336.7	>8131.8	408.0 a	8966.1 a	22.4 b	23.1
LYLE	>336.7	<8131.8	422.8 a	7295.1 b	17.9 c	22.0
LYHE	<336.7	<8131.8	269.1 b	7342.2 b	28.6 ab	28.3

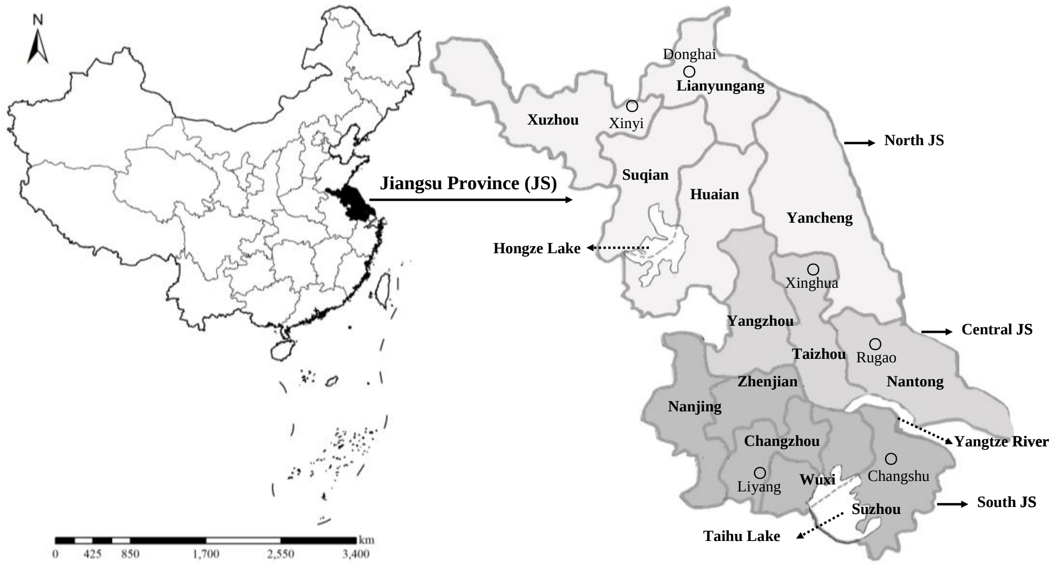

Extended Data Figure 1 | Distribution of experimental sites (open circles) in three production regions (grey shading) of south, central and north Jiangsu, China. The software ArcGis 10.2 and Photoshop CS6 were used to create the map[s].

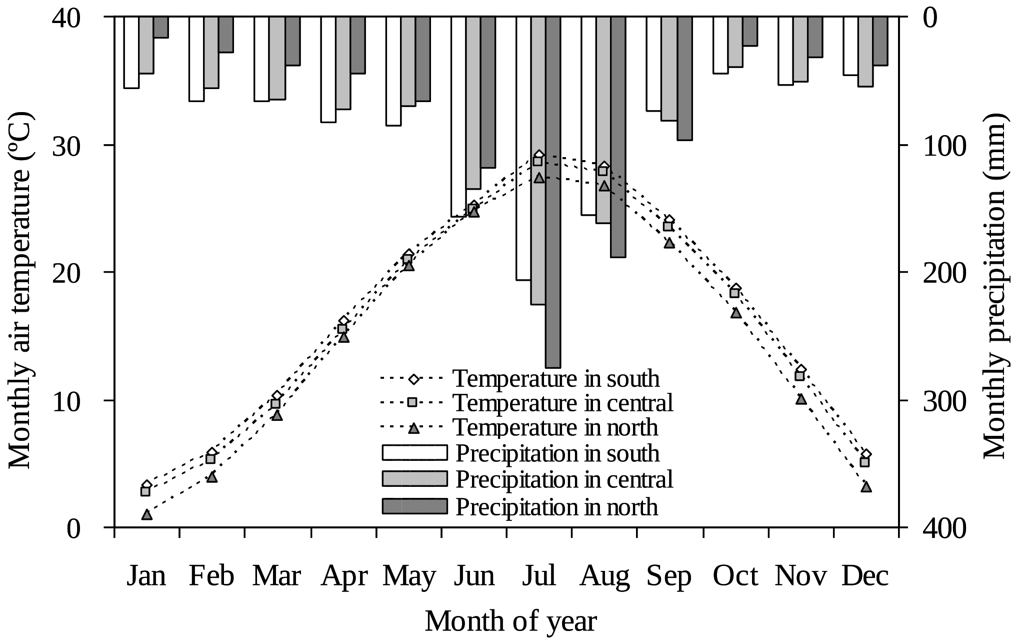

Extended Data Figure 2 | The mean monthly temperature and precipitation in south, central and north Jiangsu during the period from 2001-2013.

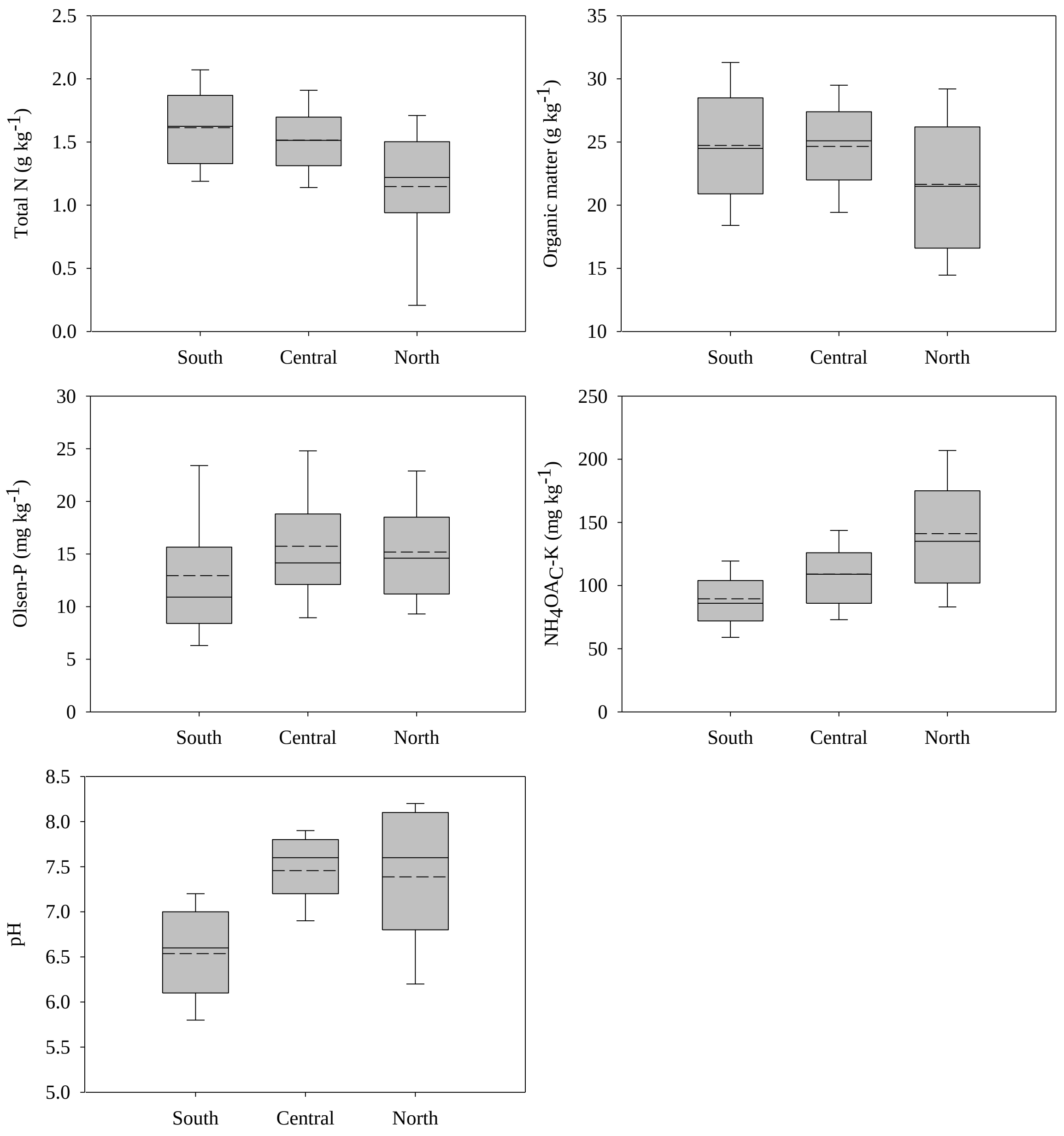

Extended Data Figure 3 | The distribution of the physical and chemical properties of the basal soil in south, central and north JS (n=1050-1350).

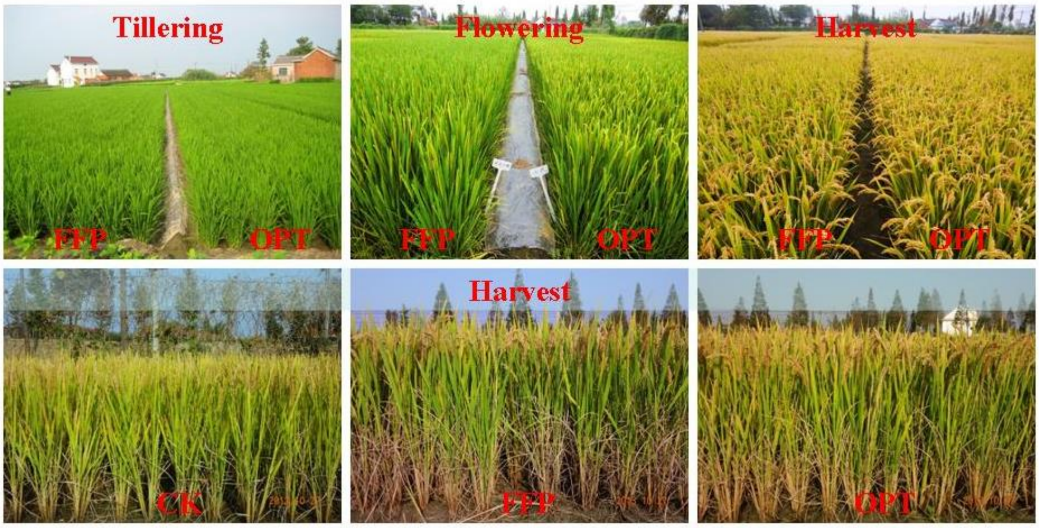

Extended Data Figure 4 | Field images of N managements at different rice growth stages in Rugao sites in Jiangsu. CK: free-N control; FFP: farmers' fertilizer practices; OPT: optimal N managements.

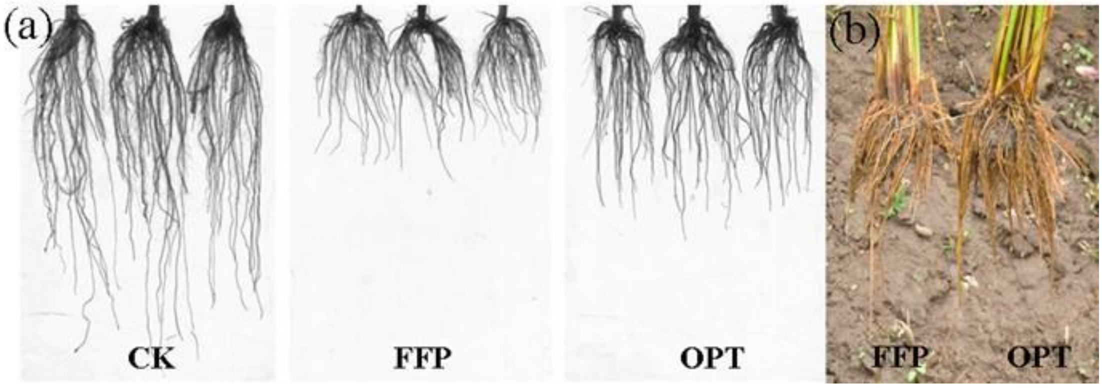

Extended Data Figure 5 | Root morphology images of N managements at the rice seedling stage 10 days after transplanting (a) and harvest (b) stages in Rugao sites in Jiangsu. CK: free-N control; FFP: farmers' fertilizer practices; OPT: optimal N managements.
